# 2243 Perceptions of translational science among faculty researchers: A survey to inform the efforts of a multidisciplinary education and research program

**DOI:** 10.1017/cts.2018.219

**Published:** 2018-11-21

**Authors:** Gayathri Devi, Jennifer C. McMains, Stephanie A. Freel, Jeffrey Hawley

**Affiliations:** Duke University

## Abstract

OBJECTIVES/SPECIFIC AIMS: Opinions regarding translational science vary incredibly. We aimed to gather a baseline of perceptions, barriers, and needs for translational science among faculty investigators. We will use these data to define areas in which the Duke Multidisciplinary Education and Research in Translational Science program (MERITS) can work to address, educate and improve. METHODS/STUDY POPULATION: Data was collected via a scalar, multiple-choice, open-ended survey including questions regarding, definition, impact, barriers, resources, and training preferences specific to translational science. Digital survey links were emailed to Duke University faculty. RESULTS/ANTICIPATED RESULTS: In total, 350 responses were collected. While perceptions of translational science varied, common defining elements were noted, including multidisciplinary collaboration (69%) and transitions between research stages (63%). Translational science was said to have an overall positive impact, despite 37% of participants stating issues of insufficient institution-wide support and 62% citing minimal training in translational science skills. DISCUSSION/SIGNIFICANCE OF IMPACT: Effective support for translational science requires a multi-faceted approach, as perceptions differ among investigators and between career stages. Duke MERITS will seek to standardize education and support ranging from teambuilding to entrepreneurship, and to promote support from institutional leadership to reduce barriers and facilitate acceleration of translational science.

